# Does ought imply can?

**DOI:** 10.1371/journal.pone.0175206

**Published:** 2017-04-12

**Authors:** Miklos Kurthy, Holly Lawford-Smith, Paulo Sousa

**Affiliations:** 1 Department of Philosophy, University of Sheffield, Sheffield, United Kingdom; 2 Institute of Cognition and Culture, Queen’s University Belfast, Belfast, United Kingdom; University of Vienna, AUSTRIA

## Abstract

Most philosophers believe that a person can have an obligation only insofar as she is able to fulfil it, a principle generally referred to as “Ought Implies Can”. Arguably, this principle reflects something basic about the ordinary concept of obligation. However, in a paper published recently in this journal, Wesley Buckwalter and John Turri presented evidence for the conclusion that ordinary people in fact reject that principle. With a series of studies, they claimed to have demonstrated that, in people’s judgements, obligations persist irrespective of whether those who hold them have the ability to fulfil them. We argue in this paper that due to some problems in their design, Buckwalter & Turri’s conclusions may not be warranted. We present the results of a series of studies demonstrating the problems with their design and showing that, with an improved design, people judge that obligation depends on ability after all.

## Introduction

The concept of obligation constitutes a fundamental component of social and moral cognition [1–3]. Although there is considerable cultural variability in terms of how people understand the content, source, and ground of obligations, anthropological evidence indicates that all human societies deploy the concept of obligation to organise human action and interaction [4, 5]. Obligations are deemed constraints that motivate social and moral behaviours. They are also deployed to understand and evaluate these behaviours. In particular, the non-fulfilment of an obligation is thought to constitute wrongdoing and may warrant blame. Thus, it is difficult to overestimate the importance of studying the folk concept of obligation.

According to a distinguished philosophical tradition that dates back at least to Kant, obligations are in force only when the persons holding them are able to fulfil them [6] (although the depth of Kant’s commitment to the principle is debated [7]). This idea, which has been accepted by most moral philosophers, albeit in a number of different prescriptive and descriptive guises [8–10], is now widely known as the principle that “Ought Implies Can” (henceforth, “OIC principle”). (The OIC principle is often discussed in terms of its equivalent contraposition “Inability Eliminates Obligation”, and much of our discussion will follow this trend).

Given the apparently quotidian belief that one cannot be constrained to do what one is unable to, it is plausible to suppose that ordinary people would make judgments that are consistent with the OIC principle, namely, that they would accept that one is not obligated to do what one is unable to. However, in a paper titled “Inability and Obligation in Moral Judgment” [11] (henceforth, IOMJ), Wesley Buckwalter and John Turri have recently presented evidence that in fact ordinary people make judgments inconsistent with the OIC principle (see also [12], and discussion in [13]). In their studies, participants had to read stories in which a person is under an obligation but is subsequently described as unable to fulfil it. (IOMJ was also interested in probing whether people have more difficulty in perceiving inability when the source of the inability is mental rather than physical, e.g. due to clinical depression. Since this issue is tangential to the OIC principle, we leave it completely aside in this paper.) For instance, participants in one study were asked to consider a case in which an agent (“Walter”) promises to pick his friend (“Brown”) up from the airport (the promise creating the obligation) but later becomes involved in a car accident and thereby rendered physically unable to keep the promise. Participants were then presented with the OIC probe, asking them to choose one of the following (randomly sequenced) statements:

1. Walter is obligated to pick up Brown at the airport, but Walter is not physically able to do so.
2. Walter is not obligated to pick up Brown at the airport, and Walter is not physically able to do so.
3. Walter is obligated to pick up Brown at the airport, and Walter is physically able to do so.
4. Walter is not obligated to pick up Brown at the airport, but Walter is physically able to do so.

In this and other scenarios—varying *inter alia* the source of the obligation involved (e.g., a promise or a social role), the type of inability (e.g., a physical restriction or a constraining feature of the environment), and the seriousness of the consequences of the obligation not being fulfilled (minor or fatal)—participants overwhelmingly chose the first option: “obligated, but not able”. On the face of it, this choice contradicts the OIC principle, since it attributes to the individual both an obligation and the inability to fulfil it.

Moreover, to confirm that participants understood the situation as involving a literal inability to fulfil the obligation, the studies in IOMJ included, after the OIC probe, an inability-comprehension probe, asking subjects whether the person under the obligation was literally unable to fulfil it. The great majority of participants confirmed that there was literal inability, and eliminating the few participants who denied literal inability did not change the general pattern of the results reported in IOMJ. Thus, IOMJ concludes with the claim that “commonsense moral cognition rejects the principle that ought implies can” [11].

The studies in IOMJ testing whether ordinary people make judgments consistent with the OIC principle also included, after the inability-comprehension probe, a blame probe, investigating whether participants would consider the individuals in their stories blameworthy for not fulfilling their obligations. They found that the great majority of participants denied that the individual is to blame in this respect, and suggested on the basis of this finding as well as the results of a separate study focusing directly on the relation between blame and inability that, for ordinary people, “Blame Implies Can”. It is important to note that the traditional view of the relation between blame and obligation as far as inability is concerned is that the presence of an inability undermines blame by eliminating the perception of wrongdoing—in particular, by eliminating the perception that someone did something wrong in not fulfilling her obligation because in fact the obligation was cancelled by the inability [14, 15]. Therefore, given that the above results indicate that the presence of an inability undermines blame without cancelling the obligation (and hence without eliminating wrongdoing), IOMJ also suggests that the traditional view of the relation between blame and obligation does not appropriately describe the relation between these concepts in ordinary cognition, and may be an invention of philosophers trying to “validate excuses” [16].

In this paper, we question the implication of the results reported in IOMJ with new evidence based on the same scenarios of inability. We argue first that there are crucial problems with the design of IOMJ’s studies. Then, we report two studies indicating the main problem with this design—namely, it does not seem to provide an appropriate test of whether ordinary people reason in line with the OIC principle. Next, we provide an overview of our new studies with an improved design. After that, we report four studies showing that the great majority of participants make judgments compatible with the OIC principle and with the traditional view of the relation between blame, obligation, and wrongdoing. Finally, we summarize our results and discuss some broader issues, such as the type of reasoning involved in participants’ judgments, the extent to which our results might generalize to cases involving culpable inability, and a possible deflationary explanation of our results in terms of excuse validation.

## Potential problems with IOMJ’s design

Aspects of IOMJ’s design, in particular the way in which the list of options of the OIC probe are framed, may make the option “obligated, but not able” the sole plausible answer, though in a way that is not inconsistent with the OIC principle.

The stories in IOMJ are characterized by an individual under an obligation who is eventually described as unable to fulfil the obligation. There is an obvious but trivial sense in which each story, taken as a whole, involves both an obligation and an inability. The inability creates tension with the expectation of fulfilment generated by the obligation. The option “obligated, but not able” matches this description of the story as a whole, while the other options do not, since they either exclude an obligation (“not obligated, and not able”) or include the ability to fulfil the obligation (“obligated, and able”; “not obligated, but able”). Moreover, the option “obligated, but not able” has an ordinary temporal reading (i.e., “obligated, but *subsequently* not able”) that mirrors the temporal narrative of the story (i.e., an obligation is made salient early in the story, then later an inability is made salient). This, too, renders the option “obligated, but not able” the best description, because it captures the temporal dimension of the contrast involved in the story as a whole.

In sum, according to our interpretation, when participants choose the option “obligated, but not able”, they are not saying that the person is *still* under the obligation even when there is an inability to fulfil it. That would be inconsistent with the OIC principle. Rather, they are saying that the stories involve a contrast between a presumed obligation (made salient first) and an inability to fulfil the obligation (made salient second). This is not inconsistent with the OIC principle, because it may well turn out that the subjects of IOMJ’s studies would accept the obligation for as long as they think there is ability, and reject the obligation after the inability is made evident in the story.

There is another aspect of IOMJ’s design that may have contributed to the problem we have outlined, and consequently to the predominant selection of the “obligated, but not able” option. The instruction for the OIC probe (“choose the option that best applies”) implies that there is a factually correct alternative among the options, and may suggest to participants that they are being tested on whether they interpreted the story correctly (as if the OIC probe had the same type of function as the inability-comprehension probe—the second probe of their design described earlier). If participants understood the OIC probe in this way, then rather than providing their personal opinion on the relation between the concepts of obligation and inability, they would simply provide the *best description* of what is involved in the story as a whole, which is plausibly the option “obligated, but not able”, as discussed above.

Finally, it is important to note that none of the stories in IOMJ explicitly state the obligation at stake in the story. In the promise scenario, the story says only that someone makes a promise; in their social-role scenarios, it says only that someone has a social role (e.g., that of a lifeguard); in another scenario, it simply describes a situation in which a small child is drowning and there is a stranger around who could easily help the child. Thus, the participant has to infer from the information given in the initial part of the story (i.e., from the fact that someone made a promise, that someone has a social role, or that someone could easily help) the existence of the corresponding obligations (i.e., the obligation to keep the promise; the obligation related to the social role; the obligation to help the drowning child). True, these inferences are somewhat obvious, and the fact that the obligations are left implicit in the stories is not a problem in itself. However, given the aforementioned problems, it may well be that at least some participants took the OIC probe to be a test on whether they believe that the initial situation described in the story entails an obligation, and chose the first option to confirm that they indeed believe that there is an obligation involved in the story.

## Study 1

In this study, we test our main claim about what lead the great majority of subjects in IOMJ’s studies to choose the “obligated, but not able” option. As we discussed above, we claim that there is an obvious sense in which the option “obligated, but not able” is the correct answer in the context of IOMJ’s design because of two main factors: (i) the option describes the fact that each story as a whole involves a contrast between an obligation and an inability to fulfil the obligation, and (ii) the option mirrors the temporal narrative of each story (i.e., an obligation is made salient early in the story, then later an inability is made salient).

Two predictions follow from our claim. First, there would be a substantial reduction of “obligated, but not able” responses in the results if one were to simply replace the connectives “but” and “and” in the original options with connectives that more clearly convey the main point of the OIC probe (i.e., that make participants focus on whether there is an inferential relation between the concepts of obligation and ability). Second, there would also be such a reduction if one were simply to invert the order of the obligation and inability clauses of the original options (e.g., changing “obligated, but not able” to “not able, but obligated”), thus creating a mismatch between the order of the clauses and the temporal narrative of the story. Our first study tests these predictions.

### Method

#### Participants

Participants were 123 adults (56 female, 67 male; *M*_*age*_ = 36.84; *SD* = 11.19; range = 52; 98% reporting English as their first language). Our data collection methodology was similar to that employed in IOMJ. In all studies to be reported in this paper, participants were recruited, tested and compensated online. We used Amazon Mechanical Turk and Qualtrics as the online platforms. All participants were U.S. residents. Each participant was paid $0.50 for approximately 4 minutes of their time. Following IOMJ, in all studies we collected around 40 responses per condition. Participants were allowed to participate in only one of the studies (or conditions) reported in this paper.

Our research design, including the procedure for informed consent, was reviewed by the Research Ethics Committee of the School of History and Anthropology at Queen’s University, Belfast, UK and by the Research Ethics Committee of the University of Sheffield, UK. Written informed consent was obtained from all participants in all of the studies reported in this paper.

#### Design, materials and procedure

The study used IOMJ’s original design of the “Walter promise” scenario (IOMJ, Experiment 1, Physical condition), but without the question asking whether Walter is to blame, and crucially, with three types of between-subjects OIC probes: the original four options of IOMJ’s design as described in the introduction (Original condition); four options using “even if” and “because” as connectives, instead of “but” and “and” (Inferential relation condition); and the original four options with the order of the obligation and inability clauses inverted (Inverted order condition). The OIC-inconsistent and OIC-consistent options of the Original, Inferential relation, and Inverted order conditions were as follows (for the sake of simplicity, we leave aside the two options where Walter was described as able to fulfil his obligation):

1. (*Original*) Walter is obligated to pick up Brown at the airport, but Walter is not physically able to do so.
  (*Inferential relation*) Walter is obligated to pick up Brown at the airport, even if Walter is not physically able to do so.
  (*Inverted order*) Walter is not physically able to pick up Brown at the airport, but Walter is obligated to do so.
2. (*Original*) Walter is not obligated to pick up Brown at the airport, and Walter is not physically able to do so.
  (*Inferential relation*) Walter is not obligated to pick up Brown at the airport, because Walter is not physically able to do so.
  (*Inverted order*) Walter is not physically able to pick up Brown at the airport, and Walter is not obligated to do so.

### Results

**Fig 1:**
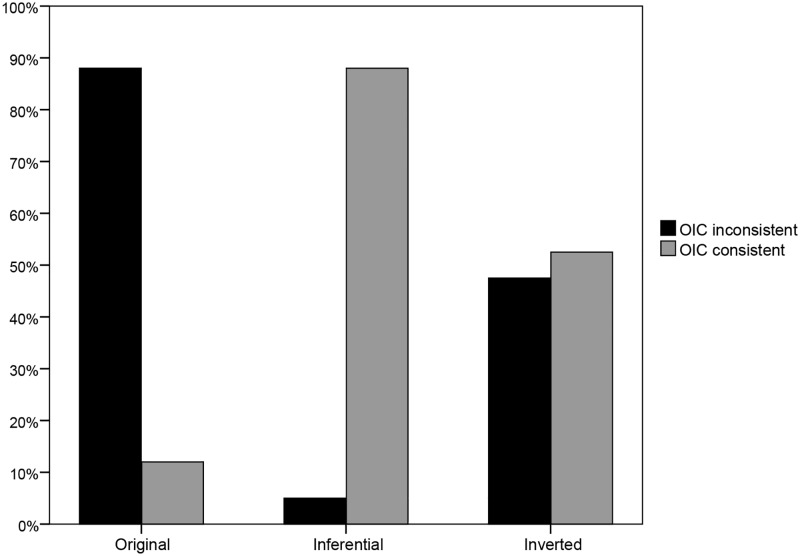
Percentage of responses consistent or inconsistent with the OIC principle in each of the three conditions.

The results of this study are shown in Fig 1. In the Original condition, we replicated the results reported in IOMJ: 88% chose “obligated, but not able”, while only 12% chose “not obligated, and not able” (*N* = 41). In the Inferential relation condition, we completely reversed the results of IOMJ: only 5% chose “obligated, even if not able”, while 88% chose “not obligated, because not able” (*N* = 42; 7% chose the remaining two options where Walter is described as able). Finally, in the Inverted order condition, the two relevant options were equally chosen: 47.5% chose “not able, but obligated” and 52.5% chose “not able, and not obligated” (*N* = 40).

Confirming that there was a substantial reduction of the obligated/unable type of response in the Inferential and Inverted conditions, Chi-square tests (with obligated/unable responses coded as “1” and the remaining responses coded as “0”) show that these conditions differed significantly from the Original condition: χ^2^ (1, 83) = 47.82, *p* < .01, φ = .76, for Inferential versus Original; χ^2^ (1, 81) = 15.09, *p* < .01, φ = .43, for Inverted versus Original.

### Discussion

The results of this study are consistent with our main claim about a problem with IOMJ’s design—the subjects choosing the option “obligated, but not able” do not interpret it in a way that is inconsistent with the OIC principle. If this option had been interpreted in terms of *obligated at the time of the inability*, the order of the clauses should not have mattered. It is also worth pointing out that the justifications following selections of the “obligated, but not able” response in the Original condition strongly suggest that, with this option, the participants were merely acknowledging that the story involved both an obligation and an inability, irrespective of whether the obligation is perceived to be in force subsequent to the onset of the inability. The majority of participants provided justifications such as:

“[Walter] promised that he would pick Brown up at the airport, which gives him an obligation to pick him up, but he was injured in a serious car accident and is therefore unable to do so.”

“He has committed to do it, and Brown is depending on him. However due to the car accident he won’t be able to make it.”

“He is obligated because he promised but he is unable to because of the accident.”

“He agreed to do it but he subsequently became physically unable.”

“He promised that he will pick up Brown at the airport. He was in an accident so he is unable to actually carry out the task.”

Finally, the results of the Inferential relation condition, using connectives that arguably make the point of the OIC probe more salient, suggest that ordinary people reason in a way that is consistent with the OIC principle, at least in this type of scenario.

## Study 2

Although our previous study suggests that the design of IOMJ is problematic in the way we discussed, one could still argue that (a) the Inferential relation condition merely distorted the results due to a different logical framing of the options, that (b) the results of the Inverted order condition do not establish *directly* that the response options of IOMJ’s design fail to test whether participants reject the OIC principle, and that (c) the qualitative justifications to the “obligated, but not able” option of the Original condition do not establish *conclusively* that this option is understood in a way that is compatible with the OIC principle. In Study 4 below, we demonstrate that the inferential relation framing does not distort the results. In this study, by asking a follow-up question regarding the relevant response options of IOMJ’s design, we provide more direct and conclusive evidence indicating that this design is problematic in the way we claim.

### Method

#### Participants

Participants were 43 adults (17 female; *M*_*age*_ = 32.84; *SD* = 8.47; range = 34; 100% reporting English as their first language).

#### Design, materials and procedure

This study was also based on the story about Walter’s promise with one crucial addition. We asked a clarificatory follow-up question in relation to the options “obligated, but not able” and “not obligated, and not able”. This follow-up question appeared on a different page, after the participant had provided a response to the original OIC probe (see previous study). Participants choosing the “not obligated, and not able” option were confronted with the following question (Question A):

You chose the option “Walter is not obligated to pick up Brown at the airport, and Walter is not physically able to do so.” With this choice, do you mean that Walter is *no longer under the obligation* to pick up Brown at the airport *after* he becomes physically unable to do so? (Yes/No)

On the other hand, participants choosing the “obligated, but not able” option had to answer the following question (Question B):

You chose the option “Walter is obligated to pick up Brown at the airport, but Walter is not physically able to do so.” With this choice, do you mean that Walter is *still under the obligation* to pick up Brown at the airport *after* he becomes physically unable to do so? (Yes/No)

The order of the options (Yes/No) was randomized in both cases. Also in both cases, participants responding “no” were asked to explain their choice (“Please explain what you meant, then.”). After the follow-up question, participants answered the inability-comprehension probe (i.e. the probe asking whether Walter was “literally unable”). We did not include a blame probe in this study either.

The logic of this study is very simple. If the selection of the “not obligated, and not able” option is to be taken as consistent with the OIC principle, then participants should predominantly answer “Yes” to Question A. Concomitantly, if the selection of the “obligated, but not able” option is taken to be inconsistent with the OIC principle, then participants should predominantly answer “Yes” to Question B. To spell it out clearly: if the design and conclusions of IOMJ are sound, then we should expect affirmative answers in both cases—but crucially so in the case of Question B, as it is the high relative frequency with which participants selected the “obligated, but not able” option that was interpreted as constituting the main evidence against OIC-consistent reasoning.

### Results

The great majority of participants (74.4%) selected the option “obligated, but not able”, while only the minority (16.3%) endorsed the option “not obligated, and not able” (the remaining 9.3% chose “obligated, and able”). As in the Original condition of the previous study, and in IOMJ in general, the selection of the apparently OIC-inconsistent option (vs. all the other options collectively) is significantly above chance level—goodness of fit against chance: χ^2^ (1, 43) = 10.26, *p* < .01, φ = 0.49. The overwhelming majority (93%) agreed that Walter is literally unable to pick up Brown form the airport.

For the rest of the analysis, we exclude participants who denied literal inability. All 7 participants (100%) choosing the “not obligated, and not able” option answered “yes” to Question A, confirming that their reasoning is consistent with the OIC principle. Now, 23 out of 31 participants (74%) selecting the “obligated, but not able” option said “no” in response to Question B—goodness of fit against chance: χ^2^ (1, 31) = 7.26, *p* < .01, φ = 0.48—, indicating that, with their response, they did *not* mean that Walter is still under the obligation after he becomes physically unable to pick up Brown.

### Discussion

Since the great majority of the participants choosing the “obligated, but not able” option answered “no” to the follow-up question (Question B), our results indicate more directly and conclusively that the selection of this option does not track OIC-inconsistent reasoning. In other words, as we discussed above, the design of IOMJ does not seem to be appropriate to test whether participants reject the OIC principle. The justifications of participants who chose the “obligated, but not able” option and answered “no” to the follow-up question also support this interpretation. Some participants were emphatic that the inability annuls the obligation, suggesting that it did not even occur to them that their response could be taken as a case of obligation ascription after the accident:

“Obviously Walter is no longer obligated to pick up Brown from the airport and anyone who tries to philosophically argue the case is limited in their scope of understanding of reality. Walter agreed to pick up someone from the airport but after being severely incapacitated due to a car accident he is no longer able (or obligated) to pick up the person and he should find an alternative.”

Furthermore, many participants pointed out that, in choosing the “obligated, but not able” option, they had intended to express the view that Walter is indeed obligated, but only up to the point at which he becomes incapacitated, which confirms our criticism of IOMJ’s design:

“I meant that Walter was obligated to pick him up until he became physically unable to do so.”

“I meant that Walter was obligated to pick up Brown. However, once he was physically unable to, he was no longer obligated.”

“He WAS obligated, but cannot physically do it so the obligation is no longer on him.”

“He agreed to do it, so he is obligated once he does that. But after getting hurt, he is not still under that obligation.”

“He had agreed on picking his friend up. But when he got into a serious accident, the obligation was suspended because he was no longer in the same position to help out his friend.”

## Overview of new studies

With our first two studies, we provided strong evidence that the design used in IOMJ does not constitute an appropriate test of whether ordinary people reason in line with OIC. In the studies to follow, we utilized a design that addresses most of the aforementioned problems and makes the task much clearer and simpler for the participants. We modified IOMJ’s design in the following ways:

1. We changed some very trivial details of the stories to make it clearer to participants that the characters in the stories are unable to fulfil their obligation, and/or to avoid misinterpretations of the story.
2. We changed the instructions of the OIC probe and the inability-comprehension probe to make their different purposes obvious to participants.
3. We positioned the inability-comprehension probe before the OIC probe, that is, just after participants read the story. And in case a participant denied that the character in the story was literally unable to fulfil their obligation, we explained to the participant that in fact the character *was* unable to do so by emphasising the relevant elements of the story; then we asked the participant to assume that there was literal inability before answering the OIC probe. (In our studies, hardly any participants disagreed that the character was literally unable to fulfil their obligation and excluding these participants from the analysis changes nothing in terms of our results and conclusions.)
4. We simplified the OIC probe by reducing its four options to two: one consistent with the OIC principle, another inconsistent with it. (Note that the two eliminated options, which say that the character in the story is able to fulfil her obligation, are completely irrelevant to testing whether people make judgments consistent with the OIC principle.)
5. We phrased the two options of the OIC probe in a way that makes it clearer to participants what the point of the OIC probe is (e.g. using the connectives “because” and “even if” instead of “and” and “but”).
6. We included a justification probe asking participants to explain their OIC choice, in order to gain some qualitative insight into the reasons motivating participants’ choices. (This step was introduced after the OIC option was irreversibly selected, so there is no reason to suppose that it could interfere with the quantitative results of the OIC probe).

The great majority of the above changes should not be controversial, as they merely clarify and/or simplify the task for the participants. Although changing the connectives of the options of the OIC probe may seem controversial, in Study 4, we demonstrate that our usage of “even if” and “because” is not problematic.

Some of IOMJ’s studies are, arguably, much less central to testing the OIC principle (e.g., Experiment 7, which tests whether the difference between moral and legal obligation is relevant to the principle). Accordingly, our studies focused on those studies that are most central to the OIC principle, namely, Experiments 1, 2, 4 and 5.

## Study 3: Promise

In this study, we used our new design to test whether people make judgments consistent with the OIC principle in relation to obligations generated by promises, using the “Walter” scenario familiar from the first two studies as well as from the first experiment in IOMJ, where it was found that 80% of participants chose the “obligated, but not able” option, apparently contradicting the OIC principle. In addition, we used different ordinary expressions that are commonly thought to encode the concept of obligation (“obligated”, “duty”, “ought”), in order to see whether there is variation in judgements as a result of these.

### Method

#### Participants

Participants were 127 adults (60 female; 67 male; *M*_*age*_ = 33.95; *SD* = 11.54; range = 53; 97% reporting English as their first language).

#### Design, materials and procedure

After indicating informed consent, participants read the following story (divergences from the wording of the story as used in IOMJ are in italics):

Walter promised that he would pick up Brown from the airport. But on the day of Brown’s flight, Walter is in a serious car accident *and is hospitalized*. As a result, Walter is not able to pick up Brown at the airport.

We added “and is hospitalized” to boost the understanding that Walter is unable to pick up Brown at the airport.

Participants were then presented with the inability-comprehension probe, whose instruction and question were as follows: “First, we would like to ask you a question to check whether you understood the story. According to the story, is the following statement true?” The statement that participants had to evaluate was: “Walter is literally unable to pick up Brown at the airport because Walter is hospitalized”. If they answered “yes”, they were presented with the OIC probe. If they answered “no”, they were given an explanation indicating that Water is indeed unable to pick Brown up because his “injuries are so serious that he requires hospitalization”; then they were asked to assume that this is the case before answering the OIC probe.

The instruction and question of the OIC probe were as follows: “Now, we would like to know your personal opinion about the situation. There isn’t a correct answer here. Which statement best reflects your personal opinion about the situation?” Participants had to choose between two randomly sequenced statements, each consistent or inconsistent with the OIC principle. In order to probe participants’ judgments with different ordinary expressions that encode the concept of obligation (“obligated”, “duty” or “ought”), participants were randomly assigned to one of three phrasing conditions:

1. Under these circumstances, Walter is still obligated to (Walter still has a duty to / Walter still ought to) pick up Brown at the airport, even if he is unable to do so.
2. Under these circumstances, Walter is not obligated to (Walter does not have a duty to / it is not the case that Walter ought to) pick up Brown at the airport, because he is unable to do so.

After choosing one of the above statements, participants were asked to justify their choice: “Please explain why you marked this option”.

Finally, participants answered a blame probe, enquiring about the degree to which they believed that Walter deserved blame for not fulfilling the obligation: “To what extent is Walter to blame for not picking up Brown?” Participants answered this probe on a seven-point scale, with “1” indicating “No blame”, “4” indicating “Moderate blame”, and “7” indicating “Full blame”.

### Results

Almost everyone (98%) agreed initially that Walter was literally unable to pick up Brown at the airport. The phrasing conditions produced no effect, χ^2^ (2, 127) = .01, *p* = .99, with 100%, 98% and 100% of participants choosing the option consistent with the OIC principle in the “obligated”, “duty” and “ought” conditions respectively. Across the phrasing conditions, 126 out of 127 participants chose the option consistent with the OIC principle—goodness of fit against chance: χ^2^ (1, 127) = 123.03, *p* < .01, φ = 0.98.

Blame ratings did not differ across phrasing conditions either—*F*(2, 124) = 1.04, *p* = .36. In general, blame ratings were very low (*M* = 1.47; *SD* = 1.02), with 92 of 127 participants opting for the “1” rating (i.e., “no blame”).

### Discussion

With our improved design, we completely reversed the results of IOMJ using three ordinary expressions that are commonly thought to encode the concept of obligation, suggesting that there is no variation in judgement due to the examined terminological variation in this domain.

Participants’ justifications suggest that, actually, *none* of their answers were inconsistent with the OIC principle. Justifications of participants who chose the “not obligated” option often expressed that, given the inability, it would be unintelligible to attribute an obligation, or that it is self-evident that the obligation does not hold:

“It seems silly to say that it’s immoral to not keep a promise in extenuating circumstances like this.”

“It makes no sense to say he should do something he isn’t able to.”

“Because he is unable to do so, it is self-explanatory.”

Sometimes they even explicated the OIC principle literally or in terms of its equivalent contraposition:

“‘Duty’ assumes he will have the ability to implement his duty, just as a soldier is excused from duty when injured.”

“I think that the existence of a duty presupposes the ability to fulfil that duty. If it is impossible for that duty to be fulfilled, it does not exist.”

“If someone is unable to do something they can’t be obligated to do it.”

Now, the justification of the only participant who chose the “obligated” option suggests that, instead of making a judgment incompatible with the OIC principle, the participant simply shifted the scope of the obligation at stake:

“Walter made an agreement with full intention of keeping it and if he cannot fulfill the agreement, notice should be sent and a proxy should be appointed to carry out the agreement as specified.”

In other words, rather than maintaining that Walter is still obligated to pick up Brown at the airport even if he is unable to do so, this participant seems to be saying that even if Walter cannot pick Brown up, he is still obligated to *do something else* to improve Brown’s situation. Since our scenario leaves open the possibility that Walter could still do something else in this respect, the response of this participant does not necessarily conflict with the OIC principle (this kind of justification will show up in later studies; we will refer to it as the ‘scope-shifting problem’, because it involves participants’ changing the scope of the obligation to include new or alternative content).

Finally, the great amount of “no blame” answers plus the overall low mean of blame ratings shows that participants think that Walter’s inability eliminated his blameworthiness for not picking up Brown at the airport, which is consistent with the blame results of IOMJ. However, contrary to IOMJ’s results, our results also suggest that participants think that the elimination of blame was linked to the fact that Walter had no related obligation under the circumstances, and, consequently, to the fact that Walter did not do anything wrong in not picking up Brown at the airport. In other words, our results are more consistent with the idea that ordinary cognition is in line with the traditional view on the relation between blame, obligation and wrongdoing.

## Study 4: Playground safety worker

Social roles are normally seen as another source of obligations. In this study, we tested whether people make judgments consistent with the OIC principle in the context of an obligation entailed by the social role of a playground safety worker. The scenario we utilized corresponds to that used in the second experiment in IOMJ, where it was found that 98% (“duty” phrasing condition) and 88% (“ought” phrasing condition) of participants chose the “obligated, but not able” option, apparently contradicting the OIC principle. In addition, we tested whether the framing of our options in terms of the connectives “even if” and “because” inadvertently biased participants towards choosing the option that is consistent with the OIC principle.

### Method

#### Participants

Participants were 86 adults (40 female, 45 male, 1 “other”; *M*_*age*_ = 37.67; *SD* = 13.25; range = 53; 98% reporting English as their first language).

#### Design, materials and procedure

Participants read first the following story:

Michael is a playground safety worker. He sees some broken glass in an area where kids sometimes play barefoot. But he is stricken by a sudden *full body* paralysis *that immobilizes him to the extent that he cannot even speak*. As a result, Michael is not able to *remove* the *broken* glass.

The first two modifications of the original scenario were to boost the understanding of inability and/or to emphasize that there wasn’t anything else that Michael could have done to improve the situation (e.g., ask other people to remove the broken glass), and thus to try to avoid the scope-shifting problem identified in the discussion of Study 3. The last modification replaced the verb “pick up” with the verb “remove,” which more clearly describes the content of Michael’s obligation in this situation.

Participants were then presented with the inability-comprehension probe, which asked them to evaluate the truth of the following statement: “Michael is literally unable to remove the broken glass from the area because he is completely immobilized.” Depending on their truth evaluations, participants proceeded to the OIC probe as specified in Study 3.

The instruction and question of the OIC probe were the same as previously. Since we showed that different ordinary expressions encoding the concept of obligation do not affect the results of the OIC probe, we used only one phrasing for the statements of the probe in this study (“obligated”). However, participants were still randomly assigned to one of two conditions. In the “explicit” condition, participants had to choose between the same type of “obligated” statements of Study 3, while in the “implicit” condition these statements were presented without the inability clauses and their connectives:

1. Under these circumstances, Michael is still obligated to remove the broken glass, even if he is unable to do so (Under these circumstances, Michael is still obligated to remove the broken glass).
2. Under these circumstances, Michael is not obligated to remove the broken glass, because he is unable to do so (Under these circumstances, Michael is not obligated to remove the broken glass).

We included the implicit condition in this study because one may argue (rather implausibly in our view) that, rather than making more explicit the main point of the OIC probe, the connectives “because” and “even if” inadvertently bias participants to choose the option consistent with the OIC principle, thus distorting the results. Against this “framing” hypothesis, we predicted that there would be no effect of condition, since the fact that we asked the comprehension probe first plus the usage of “under these circumstances” and “still” already makes the main point of the OIC probe clear enough.

After answering the OIC probe, participants answered the justification probe and the blame probe, similarly to Study 3.

### Results

Almost everyone (99%) accepted initially that Michael was literally unable to remove the broken glass. There was no effect of condition, χ^2^ (1, 86) = .387, *p* = .53, with 88% and 84% of participants choosing the “not obligated” response in the explicit and implicit conditions, respectively. Thus, altogether, the overwhelming majority of participants (86%) believed that Michael did not have an obligation under the circumstances—goodness of fit against chance: χ^2^ (1, 86) = 44.69, *p* < .01, φ = .72).

Blame ratings remained low (*M* = 1.79; *SD* = 1.41), with 59 of 86 participants opting for “no blame”. A 2(condition) x 2(OIC option choice) between-subjects ANOVA on blame scores revealed a main effect of option choice, *F*(1, 82) = 35.6, *p* < .01, η_p_^2^ = .303, but no main effect of condition (*p* = .17) or interaction (*p* = .30). Thus, participants who chose the “obligated” option saying that Michael was obligated to remove the glass blamed him more (*M* = 3.67, *SD* = 1.67) than participants who chose the option that he was not obligated (*M* = 1.49, *SD* = 1.11). Accordingly, there was a significant correlation between option choice and blame ratings: *r*_pb_ = .53, *p* < .01.

### Discussion

Once again, we completely reversed IOMJ’s results. Furthermore, as we predicted, whether the OIC options involved the inability clauses and their connectives did not affect which option was chosen. This indicates that an argument according to which the effect observed in Study 3 depends on our specific framing of the options, and, in particular, on the usage of the connectives “even if” and “because”, is not plausible. Indeed, our results provide corroboration for our contention that it is IOMJ’s design (rather than ours) that systematically distorts the results.

Justifications for “not obligated” responses again showed that participants’ responses were consistent with the OIC principle. In contrast, the justifications of the “obligated” responses (12 in total) were more varied and, overall, did not clearly indicate that these responses were incompatible with the OIC principle. Evincing the scope-shifting problem discussed in Study 3, some participants seem to have shifted the scope of the obligation to the idea that Michael still has the obligation to do (or try to do) something else to improve the situation:

“He has the job of playground safety worker, and he has been presented with an unsafe condition. If he can’t remove the glass, he should call out to the kids to avoid the area, call out to another adult, or make some kind of effort to communicate the hazard.”

“In some way if he knows there’s broken glass and no one else is notified, there needs to be a way he can communicate with someone he can or warn the kids about it.”

Since these participants seem to have misinterpreted our scenario in that they still envisaged that Michael could do something else, like informing other people, to improve the situation (or since the description of our scenario does not rule out the possibility that Michael could at least make an effort to improve the situation), their “obligated” responses are not incompatible with the OIC principle.

Some participants seem to emphasize that Michael still has the obligation to remove the glass, not at the time of his paralysis but rather as soon as he recovers:

“Well Michael may be unable to physically remove it himself, but he is obligated to do so in the sense that he should remove it as soon as possible.”

“(…) Of course if his condition worsens or doesn’t let up then he cannot act on his obligation so he won’t clean up the glass, but with the knowledge he should do it, if he can.”

This type of justification suggests that in fact these participants accept the OIC principle.

Many participants seem to appeal to the connection between the obligation and the nature of Michael’s social role (note that the word “responsibility” is often used in the sense of obligation related to a social role [17, 18]):

“It is still his responsibility as a playground safety worker.”

“That’s his job.”

“It’s his property. It’s his responsibility to get it cleaned up even if he can’t do it himself.”

“I believe as a worker and having knowledge makes you responsible.”

From these justifications, one may take that these participants indeed reject the OIC principle—the participants seem to believe that obligations related to social roles continue to be in force independent of the circumstances, and hence seem to accept that Michael is still obligated to remove the broken glass in that situation of inability.

However, it is still possible that these participants answered “obligated” simply to emphasize the obligations that are normally entailed by social roles, without necessarily rejecting the OIC principle. Because social roles are deemed to entail obligations, there is a sense in which the entailed obligations do not disappear in cases of inability, since the social role does not disappear with the inability (a playground safety worker does not cease to be a playground safety worker just because he is unable to fulfil his role in a specific situation). Accordingly, people may make a distinction between obligations that are normally entailed by a social role, and obligations that are in force at a specific point in time. This would make it possible for a playground safety worker *qua* playground safety worker to have an obligation to remove the broken glass, and yet this particular *paralysed* playground safety worker to not have that obligation. Thus, the above participants may be interpreting and answering the OIC probe simply in terms of the obligations that are normally entailed by a social role, in which case their responses are not necessarily inconsistent with the OIC principle, given that this principle has generally been assumed to be concerned with whether an obligation is still in force at the time of the inability. (It is important to note that this issue, which may have also prompted participants to choose the “obligated but not able” option in the related studies of IOMJ, is different from the main criticism we delineated concerning the way this option is framed: even in the sense of a social-role obligation being in force, there is a trivial sense in which an obligation is involved in the story and leads one to choose the option “obligated but not able”.)

Finally, the large number of “no blame” answers and low mean of blame ratings, along with the positive correlation between these ratings and OIC responses (i.e., more blame, more “obligated” response) is more consistent with the idea that ordinary cognition is in line with the traditional view of the relation between blame, obligation, and wrongdoing.

## Study 5: Lifeguard

In this study, we tested whether people make judgments consistent with the OIC principle, again in the context of an obligation entailed by a social role, but this time that of a lifeguard. While studies 3 and 4 involved an “internal” inability coming from physical restrictions, this study involves an “external” inability coming from constraints of the environment like distance in space. Furthermore, while studies 3 and 4 involved relatively minor consequences like not being picked up at the airport or stepping on broken glass, this study involves a life-and-death situation. The scenario we utilized corresponds to the one in IOMJ’s fourth experiment, where it was found that 93% of participants chose the “obligated, but unable” option that apparently contradicts the OIC principle.

### Method

#### Participants

Participants were 42 adults (11 female, 31 male; *M*_*age*_ = 38.98; *SD* = 13.13; range = 49; 98% reporting English as their first language).

#### Design, materials and procedure

Participants read the following story:

Jessica is the only lifeguard at a remote ocean beach. Two struggling swimmers are about to drown, *and no one else is around except Jessica*. She rushes in to save them, but because of the *great* distance between the swimmers, it is physically impossible for her to rescue both swimmers. Jessica rescues one swimmer but not the other.

The main modifications of the original scenario were again introduced in order to boost the understanding of inability and/or to emphasize that there wasn’t anything else that Jessica could have done to improve the situation (e.g., ask for additional help). (Other minor stylistic modifications, not indicated here, were also introduced to improve readability).

The rest of the procedure was exactly the same as in studies 3 and 4: inability-comprehension probe (“Jessica is literally unable to rescue both swimmers because they are too far apart”); OIC probe with justification probe; blame probe. In this study, there was only one OIC probe condition, with the following options:

1. Under these circumstances, Jessica is still obligated to rescue both swimmers, even if she is unable to do so.
2. Under these circumstances, Jessica is not obligated to rescue both swimmers, because she is unable to do so.

### Results

Almost everyone (95%) agreed that Jessica was literally unable to save both swimmers. The great majority (79%) of participants felt that the agent was not obligated to save both swimmers—goodness of fit against chance: χ^2^ (1, 42) = 13.71, *p* < .01, φ = 0.57.

Blame scores remained relatively low (*M* = 1.67; *SD* = 1.18), with 28 of 42 participants opting for “no blame”. However, in contrast with the previous study, participants choosing the “obligated” option did not ascribe significantly more blame to Jessica than participants choosing the “not obligated” one: *t*(40) = 1.64, *p* = .21, *d* = .49 (“obligated”: *M* = 2.11; *SD* = 1.45; “not obligated”: *M* = 1.55; *SD* = 1.09). Accordingly, there was no significant correlation between option choice and blame ratings: *r*_pb_ = .19, *p* = .21.

### Discussion

Yet again, in sharp contrast to the findings in IOMJ, the “not obligated” option was clearly preferred, even in a case in which the consequences are severe (the death of a swimmer).

Moreover, again, while the justifications of the “not obligated” responses show that these responses were consistent with the OIC principle, the justifications of “obligated” responses (9 in total) did not clearly indicate that these responses were incompatible with the OIC principle.

The great majority of “obligated” responses evinced the scope-shifting problem, in this case insisting that Jessica had a further obligation to *try to* save both swimmers:

“Even if she thinks and it would be physically impossible, she should still make as much of an effort as possible to try to save both swimmers.”

“She should still make an attempt to do whatever she can do.”

“It is her employment obligation to at least attempt to rescue both. One at a time.”

“She should at least try to save them since we don’t know if she can fail or not.”

“It is her duty as a lifeguard to do the best she can with what she has. Despite her being unable to rescue both people, she has to be moral enough to try to save both.”

Since our scenario does not rule out the possibility that Jessica can try to save both swimmers, these justifications show that the related responses are not incompatible with the OIC principle.

Again, some participants seemed to appeal to the connection between the obligation and the nature of Jessica’s social role:

“The conditions of the rescue could change however her job as a lifeguard does not change”

“She was the only one there, it was her job.”

As we discussed in Study 2, these justifications may indicate real rejection of the OIC principle. Alternatively, similarly to what we suggested, they may indicate that, with their “obligated” response, the participants are simply emphasising the defeasible obligation that is entailed by the social role of a lifeguard, without yet accepting that the obligation was in force in that specific situation—that is, without rejecting the OIC principle.

Finally, although the positive correlation between blame ratings and OIC option choices was not statistically significant, the large number of “no blame” answers and low mean of blame ratings are still more consistent with the view that ordinary cognition aligns with the traditional view of the relation between blame, obligation, and wrongdoing.

## Study 6: Drowning child

Our first three studies featured obligations created either by the agent through a social action (a promise), or by the social role of the agent (safety worker, lifeguard). In this final study, we feature a case in which the obligation does not come from a promise or a social role, but from the situation—a drowning child creating an obligation to help. The scenario corresponds to that in a particular condition (“recent”) of IOMJ’s fifth experiment, where it was found that 88% of participants chose the “obligated, but unable” option that apparently contradicts the OIC principle.

### Method

#### Participants

Participants were 41 adults (12 female, 29 male; *M*_*age*_ = 37.29; *SD* = 12.00; range = 42; 100% reporting English as their first language).

#### Design, materials and procedure

Participants first read the following story:

Michael is relaxing in the park near a pond when he sees a small girl fall in. She is drowning and definitely will die unless someone quickly pulls her out. This part of the park is secluded and Michael is the only person around. But Michael is stricken by a sudden *full body* paralysis. As a result, Michael is not able to save the girl.

We used “full body paralysis” instead of the original “leg paralysis” on the premise that this phrasing would be perceived as more of an incapacitating condition, and also as an attempt to preclude the scope-shifting problem (in a pilot study using the scenario with “leg paralysis”, a participant with an “obligated” response suggested that Michael should “at least try to crawl to save the girl”).

The rest of the procedure was the same as in the previous studies: comprehension probe (“Michael is literally unable to save the small girl because he is completely paralyzed”); OIC probe with justification probe; blame probe. As in Study 5, there was only one OIC probe condition, with the following two options:

1. Under these circumstances, Michael is still obligated to save the small girl, even if he is unable to do so.
2. Under these circumstances, Michael is not obligated to save the small girl, because he is unable to do so.

### Results

Almost all participants (98%) agreed that Michael was literally unable to save the girl. The great majority of participants (73%) thought that Michael was not obligated when there was an inability to fulfil the obligation—goodness of fit against chance: χ^2^ (1, 41) = 8.80, *p* < .01, φ = .46.

Although “no blame” was still the modal rating (18 out of 41 participants), blame scores were noticeably higher in this study (*M* = 2.73; *SD* = 2.1). For example, a *t*-test revealed that the blame scores in Study 5 and Study 6 differed significantly, *t*(61) = 2.84, *p* < .01, *d* = 0.72 (equality of variances not assumed). Moreover, a *t*-test showed that, similarly to Study 4 (but unlike in Study 5), blame scores were significantly higher for participants choosing the “obligated” option than for those choosing the “not obligated” option: *t*(39) = 5.15, *p* < .01, *d* = 1.65 (“obligated”: *M* = 4.91; *SD* = 2.02; “not obligated”: *M* = 1.93; *SD* = 1.48). Finally, there was a strong, significant correlation between statement choice and blame ratings: *r*_pb_ = .64, *p* < .01.

### Discussion

We again reversed the results of IOMJ, although, of the four studies, this one had the lowest percentage of “not obligated” responses.

However, an analysis of the justifications of “obligated” responses (11 in total) suggests that this study was beset by a major problem. About half of the participants do not seem to have maintained the assumption of literal inability when answering the OIC probe, mostly because they took the full bodily paralysis to be a controllable emotional reaction (involving especially fear):

“He needs to overcome his fear and save the girl.”

“You have to overcome your fear a person’s life is at stake.”

“It was just an emotional reaction which he could overcome.”

“Michael is responsible to get control of himself and save the girl. He can control his emotion and reactions and needs to pull himself together.”

“He is responsible to save her even if he SEEMS unable to do it. I believe his perception of being paralyzed is not real.”

If these justifications indeed correspond to the reason why participants chose the “obligated” response, then their responses are not inconsistent with the OIC principle after all.

Some participants’ responses revealed the scope-shifting problem again in terms of obligation to try, which, as we already discussed, is not incompatible with the OIC principle:

“He is obligated to at least TRY. If he can’t, he can’t. Maybe the water is deep and he can’t swim. But he should at least try no matter what.”

“I have never heard of a sudden full body paralysis like this, and it seems like Michael should still be trying to help.”

A few participants emphasized that there was a (moral) obligation in the situation:

“He had a duty to act, a moral obligation. His fear paralyzed him and he was unable to act.”

“He is morally obligated to save the girl.”

“Well I assume nothing has changed about the girls [sic] situation just because Michael can’t move so the obligation to save her is still there, even if he can’t move it still exists.”

These justifications seem indeed to indicate a response that is inconsistent with the OIC principle.

The fact that the overall mean of blame ratings was a bit higher in this study (in comparison with studies 4 and 5) is not incompatible with the view that inability undermines blame, since the mean was substantially affected by the ratings of the participants with “obligated” responses that did not assume inability as shown by their justifications (with these participants eliminated from the analysis, the overall blame mean drops from “2.73” to “2.25”, which is much closer to, and non-significantly different from, the overall mean of studies 4 and 5). Moreover, a large number of participants still chose the “no blame” answer. Finally, these blame ratings plus the strong correlation between blame ratings and OIC choice indicate that ordinary cognition is in line with the traditional view of the relation between blame, obligation, and wrongdoing.

## General discussion and conclusion

**Fig 2:**
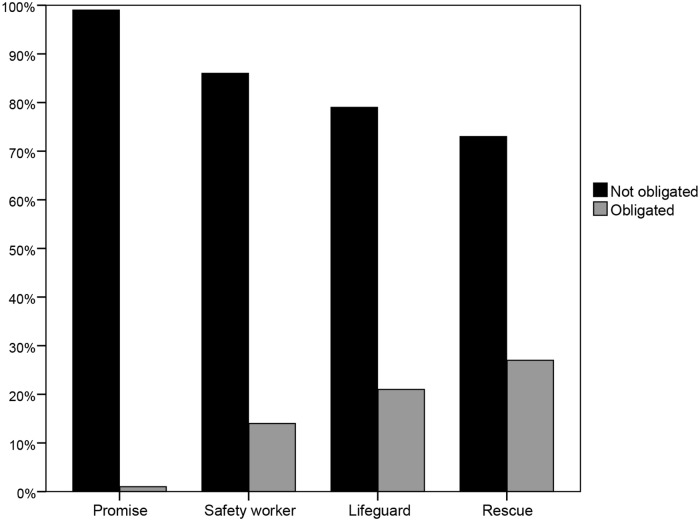
Percentage of responses to the OIC probe in studies 3, 4, 5 and 6.

In studies 1 and 2, we provided evidence indicating that there is a problem with IOMJ’s research design, namely that it does not unambiguously test whether people reason in line with the OIC principle. In the following four studies, using an improved design, we showed that the great majority of participants judge that a person is not under an obligation if she is not able to fulfil it, completely reversing the results of IOMJ (see Fig 2). Study 3 showed that the obligation to fulfil a promise is deemed annulled when the agent is not able to fulfil it. This study also indicates that this is the case irrespective of the particular term used to express the concept of obligation (“obligated”, “duty” or “ought”). Using a different scenario, Study 4 demonstrated that these results do not depend on our particular use of connectives—rather, it is the results of IOMJ that appear fragile in this respect, as also shown in Study 1. Studies 5 and 6 extended these findings to cases in which the consequences are more serious (the death of a person).

Studies 4, 5, and 6 still saw a relevant minority of participants choosing the “obligated” response, suggesting that there may be some individual variation in this domain. However, a substantial part of “obligated” responses still seems to derive from a misinterpretation of the OIC probe and/or the scenarios, as evinced by justifications demonstrating the “scope-shifting” problem, which appeared across all studies, by justifications showing that the participants did not keep the assumption of inability, which appeared in Study 6, and by justifications that seemed simply to emphasize the obligations normally entailed by social roles, which appeared in studies 4 and 5. Of course, if this is correct, it raises the question as to why there was such misinterpretation. The scope-shifting problem may be a result of participants’ inclination to blame the person specifically for not *trying* to do her best to minimize the bad consequences of the situation, something our studies did not control for. The misinterpretation of “full body paralysis” in terms of controllable emotional reaction in Study 6 may have a similar explanation. An interpretation of the OIC probe in terms of whether the obligations are entailed by the social role (instead of in terms of the entailed obligations being in force) may be difficult to avoid completely in contexts involving social roles, since this may always be a possible reading of the question.

Moreover, one may raise the question of why there may have been an increase in misinterpretation between Study 3 and Study 6 correlated with the increase in “obligated” responses. There is a sense in which the consequence of scenario 6 (the death of a small girl) is worse than that of scenario 5 (the death of an adult), which in turn is worse than that of Study 5 (the risk stepping on a broken glass), which in turn is worse than that of Study 3 (not being picked up at the airport). (A small study asking participants to rate these scenarios in terms of their seriousness confirmed this hierarchy—*N* = 25, Kendall’s W = .78, p < .01). Thus, it is also possible that this increase in seriousness may have contributed to the increase in the amount of misinterpretation from scenario 3 to 6, by pushing participants to see the situation as less determined and hence to be more hopeful about a positive outcome.

If our take on the minority responses is correct, the range of individual variation suggested by our sample is rather small—almost all participants make judgements consistent with the OIC principle in the types of scenarios that we probed. This raises two broader and complementary issues. The first issue concerns the type of reasoning involved in participants’ judgments—in particular, the type of implication connecting the concepts of obligation and ability. The second issue concerns the generalizability of our results to different types of contexts—in particular, to contexts involving culpable inability [19, 20]. We discuss these two issues in turn.

Since implication can take many forms, the general statement of the OIC principle underspecifies the nature of the inferential relationship between ‘ought’ and ‘can’. In the philosophical literature, the usual candidates for this relationship are presupposition [21], conversational implicature [22], pragmatic-logical inference [23], and conceptual or analytic entailment [10, 24]. This issue is important because each account of implication (insofar as they are understood as hypotheses about ordinary cognition) will entail different predictions about people’s judgments. For instance, because on the conceptual-entailment account the inference is logically necessary, attributions of inability (to X at time Y) would preclude attributions of obligation (to X at time Y) across all types of context. In contrast, because on the conversational-implicature account the inference is defeasible, attributions of inability would preclude attributions of obligation in some contexts but not in others. The homogeneity of our results is consistent with any of these accounts—e.g., it may be that our participants reasoned in terms of conceptual entailment or it may be that they reasoned in terms of conversational implicature, but our studies were limited to contexts where the implicature is not cancelled. Accordingly, our results raise doubts about the claims made in IOMJ, whatever interpretation of implication the authors may have in mind (Buckwalter and Turri are not explicit in their article about whether they have a specific version of the OIC principle in mind). However, some results in the literature related to contexts of culpable inability suggest that at least the conceptual-entailment account is not correct, which leads us to the second issue.

In order to probe whether ordinary people reject the OIC principle in terms of the conceptual-entailment account, Chituc et al. [19] presented participants with two types of scenarios of inability. Some scenarios (“low-blame scenarios”) were similar to those of our studies in that their main character did not have control (or had little control) over the source of the inability (e.g., one could not fulfil a promise because one’s car broke down unexpectedly). In relation to these scenarios, Chituc et al. obtained results that were overall similar to ours (i.e., the majority of participants gave responses consistent with the OIC principle), which provides further evidence in favour of our claim that IOMJ’s conclusions are problematic.

However, the other scenarios (“high-blame scenarios”) differ from those of our studies in that their main character had total control over the source of the inability (in fact, the inability was intentionally created by the character himself). In their first experiment, for example, participants were presented with the following vignette:

Adams promises to meet his friend Brown for lunch at noon today. It takes Adams thirty minutes to drive from his house to the place where they plan to eat lunch together. Adams decides that he does not want to have lunch with Brown after all, so he stays at his house until eleven forty-five. Because of where he is at that time, Adams cannot meet his friend Brown at noon, as he promised. [19]

Participants were then asked whether they agree with the statement “At eleven forty-five, it is still true that Adams ought to meet Brown at noon,” which they answered by choosing a point on a scale from -50 (“completely disagree”) to 50 (“completely agree”), with 0 as the midpoint (“neither agree nor disagree”). In this condition, 60% of participants provided a response inconsistent with the OIC principle (i.e., answered above the midpoint). Moreover, in their third experiment, they obtained a similar result using a different high-blame vignette (50% of participants provided OIC-inconsistent responses in this new condition). With these results (and others to be discussed below), Chituc et al. claim that the conceptual-entailment account cannot be correct.

Although our studies did not address high-blame contexts, we would like to make two comments about Chituc et al.’s related results. First, as the qualitative data of our studies show, participants are prone to misinterpreting the scenarios and/or the OIC probe in a way that renders their OIC-inconsistent responses of questionable value as evidence concerning whether they reject the OIC principle. Now, it is possible that this tendency to misinterpretation was even more accentuated in Chituc et al.’s high-blame scenarios, given that their cases of self-imposed inability are somewhat bizarre from the perspective of the protagonist’s behaviour (making a decision to self-impose an inability after making a promise to a *friend* without even notifying them). Thus, we believe that one has to be cautious about whether Chituc et al.’s high-blame results demonstrate that the conceptual-entailment account is incorrect (for a detailed discussion of cases of self-imposed inability from the perspective of the conceptual-entailment account, see [24]).

Second, even supposing that Chituc et al.’s studies indeed reveal that ordinary people reject the OIC principle qua conceptual entailment, it is still plausible to suppose that there is a very stable inferential relation between the concepts of obligation and ability—i.e. that the OIC implication is a core element of the set of inferential relations normally associated with the folk concept of obligation. Ordinary people seem to understand obligations as having a behavior-regulating role—i.e., obligations are deemed *social or moral constraints* on actions [2, 3]. Accordingly, it would seem rather incoherent to think that such a constraint should still be in force when it cannot be effective, namely, when the action in question cannot be carried out (for a similar argument, see [23]). Cases of self-imposed inability may simply constitute exceptions to this. A brief consideration of the literature on concepts in the cognitive sciences may help convey our main point here [25–27]. Although the inferential relation between the concepts of obligation and ability may not be analytical (à la the classical theory of concepts), it may be prototypical (à la prototype theories) and/or it may be part of a folk theory delineating the role of obligations (à la the theory view of concepts).

We turn now to the discussion of our blame results and of our perspective on how ordinary people understand the relation between blame, obligation/wrongdoing, and inability. In all our studies, a large number of participants attributed no blame to the individual for the fact that the obligation was not fulfilled. The mean blame ratings were low in all studies too. They were highest in Study 6, but this was likely due to the fact that some participants did not maintain the assumption of inability appropriately. Thus, overall, our results suggest that, for ordinary people, inability undermines blame, which is consistent with the results in IOMJ on blame. Contrary to the claim made in IOMJ that blame attributions are unrelated to obligation attributions, the low percentage of the “obligated” responses plus the correlations between blame ratings and OIC probe choices (i.e., more blame, more “obligated” responses) in our results are consistent with our hypothesis that ordinary cognition is in line with the traditional view that, in cases of inability, blame reduction is mediated by obligation/wrongdoing elimination.

However, there is another set of results in Chituc et al. [19] that apparently goes against our perspective on how ordinary people understand the relation between blame, obligation/wrongdoing, and inability—indeed, these results apparently go even against the aforementioned hypothesis that, even if not analytical, the OIC implication is a core element of the set of inferential relations normally associated with the folk concept of obligation. (This is something that is not explicit in Chituc et al.’s discussion: while some of their results, as discussed above, go against the conceptual-entailment account of the OIC implication but not necessarily against other accounts, some of their results, to be discussed next, go against a much broader range of accounts.)

In their second experiment, Chituc included only a low-blame scenario of inability (in this scenario, the character cannot keep the promise to meet with his colleague at noon because his car unexpectedly breaks down). They asked participants how much they agreed with statements saying that the character ought to keep the promise, is to blame for not keeping the promise, and can keep the promise (the same agreement scale was used, as explained before in relation to their first experiment). Restricting the analysis to participants who disagreed with the “can” statement, since these are the relevant cases for our discussion, Chituc et al. found a correlation between blame and obligation responses (*r* = .24, *p* < .01), but while they found a correlation between blame and ability responses (*r* = .24, *p* < .01), they did not find a correlation between obligation and ability responses (*r* = .07, *p* = .37). This suggests that when people give OIC-consistent responses, they are simply engaging in excuse validation [16]—i.e., they are denying obligation to be consistent with a primary reduction in blame attribution based on the situation of inability, rather than because of an inferential relation between the concepts of obligation and ability. In other words, it suggests that the relation between obligation and ability is completely mediated by blame attributions.

However, the above pattern of correlations was not replicated in their Experiment 3 in the context of its moral/unable conditions, since they did not found a correlation between blame and ability responses while observing a trend (*r* = .18, *p* = .09) between obligation and ability responses. (It is worth noting that the non-moral conditions of Chituc et al.’s Experiment 3 are completely irrelevant to our issue here, since these conditions do not involve non-moral obligations as Chituc et al. appear to claim; rather they involve what is discretionary—a decision to go to the cinema does not involve a non-moral obligation, it simply involves what is under someone’s discretion.) Furthermore, we carried out further analyses of the results of Experiment 3 (based on Supplementary Data S4 available at the publisher’s website), showing that the relevant correlations go in the direction of our picture. (The following correlations were not reported in the original article.) If one restricts the analysis to participants who disagreed with the “can” statement (thus including only those subjects whose responses are mostly relevant to our discussion), one finds that there is a correlation between blame and obligation responses (*r* = .40, *p* < .01), but while there is still no correlation between blame and ability responses (*r* = .03, *p* = .79), there *is* a correlation between obligation and ability responses (*r* = .32, *p* < .01). Thus, although we acknowledge that this is still a contentious issue, and that it is still possible that our results were prompted by an excuse-validation bias, we believe that our overall picture on the relation between blame, obligation/wrongdoing and inability remains plausible.

To conclude, our studies provide strong evidence that despite IOMJ’s claims to the contrary, people do make judgements largely compatible with the OIC principle, at least in cases in which the inability is not self-imposed. Furthermore, although we acknowledge that this question is far from settled, we believe that our results are best explained by maintaining that there exists a strong inferential relation between the concepts of obligation and ability in folk cognition. Finally, our results are consistent with the idea that ordinary reasoning is in line with the traditional view that blame reduction is related to obligation elimination via the elimination of wrongdoing.

## Supporting information

S1 Dataset (ZIP)
